# 

**Published:** 1883-02

**Authors:** 


					﻿Contributors:
Alf. L. Loomis, M.D..................New	York
Fessenden N. Otis, M.D..............
F. H. Bosworth, M.D................... “
J. L. Little, M.D..................... “
J. Williston Wright,	M.D............ “
C. E. Francis, D.D.8., M.D.S........
A. J. C. Skene, M.D...........Brooklyn,	N. Y.
O.	A. Marvin, D.D.S...........
Edwin T Darby, M.D., D.D.S......I’liila.,	Pa.
P.	8. Wales, M.D.......Surg.-Gen’l	U. S. Navy
I H. F. Campbell, M.D..............Augusta,	Ga.
C. li. Mastin, M.D., LL.D........Mobile, Ala.
J. J. Chisolm, M.D............Baltimore, Md.
C. F. Bevan, M.D..............
I.	E. Atkinson, M.D........... “
H. McGuire, M.D..............Richmond, Ya.
F. P. Porcher, M.D..........Charleston,	8. C.
Jas. T. Whittaker, M.D.........Cincinnati, O.
J.	Ransohoff, M.D., F.R.C.S....	“
F. Forchheimer, M.D................ “
A. R. Jackson, M.D..............Chicago,	Ill.
S. V. Clevenger, M.D............
E. F. INGALS, M.D...............
J. H. Carstens, M.D...........Detroit,	Mich.
PROSPECTUS
For Vol. IV., 1883, of the Independent
Practitioner.
This Joγrnal is independent of colleges,
cliques, isms or any advertising firm; there-
fore impartiality pervades its columns in the
sole interest of the Profession.
It is cosmopolitan in its circulation and
scientific resources; consequently of equal
interest to the specialist and general prac-
titioner everywhere.
That which is new and useful in Medicine,
Surgery, Obstetrics, Dentistry, Pathology
and Popular Science is given to its readers
in an intelligent and concise style.
				

